# The Village Pastor's Surgical and Medical Guide: In Letters from an Old Physician to a Young Clergyman, His Son

**Published:** 1839-04

**Authors:** 


### Art. XIII.

*The Village Pastor's Surgical and Medical Guide: in Letters from an old Physician to a young Clergyman, his Son.* By Fenwick Skrimshire, m.d.—*London,* 1838. 8vo. pp. 445.

In a former Number, (No. X. p. 381,) when reviewing several of the then recently published popular works connected with medical science, we expressed ourselves convinced that the public were benefited by works of this class, and that we therefore regarded their announcement with satisfaction. The publications that then came under our notice, however, referred either to the principles of physiology or else to the mere prevention of disease; and any approbation of works of the same general class must be much more jealously granted to such as are devoted to popular instruction in the art of curing diseases. The tendency of the former kind of books is to enlarge the mind, and induce in their readers observation on those every-day circumstances which tend to the increase or diminution of the general happiness; while the latter are placed, for the purposes of practical application, in the hands of those in whom there cannot be that balance of knowledge which may enable them correctly to appreciate many difficult conditions of the morbid state, nor that extent of information which is necessary to enable them to weigh and discriminate the relative value of opposing medical opinions. There is no one art in which the trite adage of "a little knowledge is a dangerous thing" is so applicable as in medicine. It has long been, and doubtless will continue long to be, the misfortune of many to study popular works on the treatment of disease, in order to understand maladies under which they themselves really labour, or fancy that they labour, and thus to learn a lesson which ever proves in its effects fraught with bitter results: in truth, all the treatises, without one exception, that have hitherto been published to this end are worse than useless, and well merit the pungent remark of the author of the "Doctor," that "a book which directs people how to physic themselves ought to be entitled Every Man his own Poisoner." Nevertheless, there are situations in which the experience of every day shows us that some slight but judicious acquaintance with the simpler and more obvious principles of the practice of physic may be usefully possessed by non-medical persons whose lot may have thrown them into conspicuous and responsible situations amongst a poor population, to whom medical assistance is not only difficult of access, but is often not to be obtained at all. The recent enquiry into the state of pauper medical relief has placed in strong light the fact that a very large proportion of the poor population of this country, whose employment obliges them to reside at remote distances from the residence of the medical practitioner, does not enjoy that prompt and constant professional assistance which is absolutely required under many conditions of severe malady.

It is to supply the requisite knowledge amongst educated individuals in situations such as this, that a sufficient work has been a desideratum; and, though many have been published to this purpose, yet, generally speaking, either from attempting too much, or from meagerness upon those topics where the non-professional person may be useful, such works have proved to be anything but beneficial.

There can be no doubt that it is far from an easy task to write a satisfactory work on popular medicine. There are many qualifications necessary to the individual who may undertake it: it requires a peculiar mind to compose a work for the guidance of that portion of the public who are untaught in the principles of the art, as well as ignorant of the frame on which these are to be applied. The writer should not only possess a clear and comprehensive knowledge of the principles of his profession, together with a long experience of their practical workings, but must likewise be endowed with a certain tact and acquaintance with the minds of men of liberal education, whereby he may be enabled to appreciate their caliber in respect to matters with which they are not professionally conversant. It is also especially essential that a work to this end should be written with the most jealous care; for it must be borne in mind that neither previous education nor opportunity have given the power to acquire that knowledge and experience by which to discriminate between the resembling symptoms of different diseases: whence the necessity of plainly pointing out and laying much stress upon the when and the where the non-professional person should stay his interference. The style of such a work should be concise, plain, and comprehensive, and, of all things, free from controversy; plain directions, and judicious limits to these, should be its chief characteristics.

The work which is at the head of this notice in many respects accords with these requisites. It is addressed to the most proper class of persons, and explains well and sufficiently how far the country curate may venture to extend his powers of usefulness, by administering medical relief to his sick poor; and it clearly points out those bounds, "quos ultra citraque nequit consistere rectum:" it is not burdened by details, but places in a strong point of view the general aspect of disease, and in plain terms enables the man of liberal education to appreciate where he may apply the remedies at his command.

On these grounds, were it not for some most vital imperfections, we might recommend the work of Dr. Skrimshire, not only to the class of persons to whom the author has addressed it, but likewise to the junior students of the profession, whose opportunity of seeing disease during pupilage is often much greater than is commensurate with the knowledge attained. We also fully believe that it only requires a few good popular works of this kind to put a stop to the far-spreading quackeries of the present day. Judicious and useful knowledge thus honestly acquired cannot fail to work results more hostile to the diffusion of such baneful and irrational absurdities as homœopathy, animal magnetism, &c., than any acts the legislature may devise, as well as to supersede the injurious nostrums so liberally dispensed by the officiousness of Lady Bountifuls.

We were pleased to meet, in Dr. S.'s work, many very useful hints, which, from being so apparently trifling, are omitted in larger or more pretending volumes: we allude to such little matters as the swelling of the finger upon the ring.

"Another common accident to a finger, which may be mentioned as appropriately here as elsewhere, is the accidental confinement of a ring with so much tightness as to prevent the withdrawal of it by any ordinary means. This sometimes arises from some sudden swelling of the finger; at other times, from slow and gradual growth of the finger in size, when the person has neglected to remove the ring for years. But the inconvenience arises more frequently from the person's own indiscretion in forcing on a ring that is too small, when the ineffectual attempts to withdraw it increase the evil by bringing on soreness and inflammatory swelling. In all these cases first try the effect of cold by immersing the hand in ice-cold water: if this does not alone reduce the swelling, hold the hand up so as to encourage the return of the venous blood, and at the same time apply cold by means of wet rag. If this also fails, make trial of the following ingenious device, which, I have lately been informed, very frequently proves successful. Wrap a piece of packthread closely and tightly round and round the finger, beginning at its extremity until you reach the ring; then insert the end of the string, by means of a fine blunt bodkin, or other such instrument, under and through the ring, draw it tight, and then begin to untwist the string from around the finger, and you will gradually bring down the ring at the same time. When all these means fail, the ring must be filed off, as the consequence of its retention will be ulceration and perhaps mortification." (p. 82.)

The observations upon small-pox and the relative value of vaccination (pp. 273-289) are exceedingly good: the plain, simple, and concise way in which the whole question is stated cannot fail to be of service to the good cause.

Having said thus much in favour of this little work, we are convinced that Dr. S. will take in good part our pointing out some few of the blots in its execution, which are particularly disfiguring, and which we think should induce him to hasten to revision. Under the head of Burns and Scalds, (p. 68,) though the severer applications of continued cold and turpentine are dwelt upon, the more simple and really valuable applications of cotton and flour are omitted; again, when speaking of Abscess, (p. 111,) though the difficulties of diagnosis are briefly mentioned, yet stress sufficient is not laid upon the impropriety of any but medical men interfering with them.

The use of strychnine (p. 196) is much too loosely alluded to, and too confidently recommended even for a professional work; but a remedy of a nature so frightfully powerful should have found no mention in a book devoted to popular instruction.

Some passages we met with in the course of reading this volume induce us to think that Dr. S. has not always kept pace with the learning of the day. For instance, in stating that there is no antidote to arsenic, he makes no mention of the recent trials of the tritoxide of iron, and he seems not to have read the favorable accounts that have been given of its efficacy.

Before we finish this notice, we cannot refrain from expressing our disapprobation at the covert philippic in which the author has indulged against some of the first practitioners of the day; especially against one who, as a surgeon, ranks among the very chief in England. We allude to the observations on Hysteria (p. 172). The whole paragraph is unfortunate, and we are convinced that it will be only for Dr. S. to read it in these pages to see the truth of our strictures.

> "Hysteria (anglicè hysterics) is described by medical authors as a very Proteus, imitative of almost every other disease in the nosology. Very learned physicians have written histories of its imitative powers, and have attempted to describe its anomalous varieties; and celebrated surgeons have of late gone so far as to speak and write about hysteric knees and hysteric breasts. It does not require much sagacity to foretel that all this will in a few years be deemed arrant nonsense; and to have thus huddled together an immense variety of distinct disorders under the title of hysteria will be deemed, and truly so, a proof of ignorance."

The worst part of the whole book, however, are the appendixes: in fact, in their present state, we consider the book almost unfit to be circulated amongst non-professional persons: we refer to the extreme largeness of the doses of the medicines recommended: for instance, the dose of arsenic for an adult is stated to be eight drops, for a child four; calomel for an adult six grains, for a child four; laudanum for an adult forty drops, for a child eight; wine of colchicum from forty to sixty drops for an adult, &c.

It is with much regret that these very glaring errors have forced themselves upon us. We trust, however, that Dr. Skrimshire will hasten to prevent the mischief they may occasion; especially as there really is much that is useful and excellent in these letters which he has indited to his son.

---

